# Cancer of the corpus uteri: 2021 update

**DOI:** 10.1002/ijgo.13866

**Published:** 2021-10-20

**Authors:** Martin Koskas, Frédéric Amant, Mansoor Raza Mirza, Carien L. Creutzberg

**Affiliations:** ^1^ Division of Gynecologic Oncology Bichat University Hospital Paris France; ^2^ Department of Gynecologic Oncology KU Leuven Leuven Belgium; ^3^ Center for Gynecologic Oncology Amsterdam Netherlands Cancer Institute Amsterdam The Netherlands; ^4^ Center for Gynecologic Oncology Amsterdam Amsterdam University Medical Center Amsterdam The Netherlands; ^5^ Department of Oncology, Rigshospitalet Copenhagen University Hospital Copenhagen Denmark; ^6^ Department of Radiation Oncology Leiden University Medical Center Leiden The Netherlands

**Keywords:** chemotherapy, corpus uteri, endometrial cancer, FIGO Cancer Report, gynecologic cancer, radiotherapy, surgery

## Abstract

Endometrial cancer is the most common gynecological malignancy in high‐ and middle‐income countries. Although the overall prognosis is relatively good, high‐grade endometrial cancers have a tendency to recur. Recurrence needs to be prevented since the prognosis for recurrent endometrial cancer is dismal. Treatment tailored to tumor biology is the optimal strategy to balance treatment efficacy against toxicity. Since The Cancer Genome Atlas defined four molecular subgroups of endometrial cancers, the molecular factors are increasingly used to define prognosis and treatment. Standard treatment consists of hysterectomy and bilateral salpingo‐oophorectomy. Lymphadenectomy (and increasingly sentinel node biopsy) enables identification of lymph node‐positive patients who need adjuvant treatment, including radiotherapy and chemotherapy. Adjuvant therapy is used for Stage I–II patients with high‐risk factors and Stage III patients; chemotherapy is especially used in non‐endometrioid cancers and those in the copy‐number high molecular group characterized by *TP53* mutation. In advanced disease, a combination of surgery to no residual disease and chemotherapy with or without radiotherapy results in the best outcome. Surgery for recurrent disease is only advocated in patients with a good performance status with a relatively long disease‐free interval.

## Synopsis

The latest state‐of‐the‐art treatment for endometrial cancer is described, incorporating the most recent new data that influence its clinical management.

## 1 STAGING

### 1.1 Anatomy

#### 1.1.1 Primary site

The upper two‐thirds of the uterus located above the internal orifice of the uterus is termed the corpus. The fallopian tubes enter at the upper lateral corners of an inverse pear‐shaped body. The portion of the muscular organ that is above a line joining the tubouterine orifices is referred to as the fundus.

Cancer of the corpus uteri is usually referred to as endometrial cancer, which arises from the epithelial lining of the uterine cavity. Its first local extension concerns the myometrium. Cancers arising in the stromal and muscle tissues of the myometrium are called uterine sarcomas and are not discussed in this overview (readers are directed to Mbatani et al.^1^).

#### 1.1.2 Nodal stations

The lymphatic system of the corpus uteri is formed by three main lymphatic trunks: utero‐ovarian (infundibulopelvic), parametrial, and presacral. They collectively drain into the hypogastric (also known as internal iliac), external iliac, common iliac, presacral, and para‐aortic nodes. Direct metastases to the para‐aortic lymph nodes are uncommon. This is surprising given that a direct route of lymphatic spread from the corpus uteri to the para‐aortic nodes through the infundibulopelvic ligament has been suggested from anatomical and sentinel lymph node studies.

#### 1.1.3 Metastatic sites

The vagina, ovaries, and lungs are the most common metastatic sites.

### 1.2 Rules for classification

Surgical staging of endometrial cancer replaced clinical staging by the FIGO Committee on Gynecologic Oncology in 1988 and again revised in 2009. Rules for classification include histologic verification of grading and extent of the tumor.

### 1.3 Histopathology

#### 1.3.1 Histopathologic types

Histopathologic typing should be performed using the latest WHO classification of tumors.^2^ All tumors are to be microscopically verified.

The histopathologic types of endometrial carcinomas are^2^:

- Endometrioid carcinoma: adenocarcinoma; adenocarcinoma‐variants (with squamous differentiation; secretory variant; villoglandular variant; and ciliated cell variant)
- Mucinous adenocarcinoma
- Serous adenocarcinoma
- Clear cell adenocarcinoma
- Undifferentiated carcinoma
- Neuroendocrine tumors
- Mixed carcinoma (carcinoma composed of more than one type, with at least 10% of each component).

Apart from the classification of endometrial carcinoma, carcinoma of the endometrium comprises mixed epithelial and mesenchymal tumors including:

- Adenomyoma
- Atypical polypoid adenomyoma
- Adenofibroma
- Adenosarcoma
- Carcinosarcoma: currently carcinosarcomas, in which both epithelial and mesenchymal components are malignant and aggressive tumors, are considered metaplastic carcinomas, and are treated as aggressive carcinomas.

Endometrial cancers have primarily been classified as endometrioid versus non‐endometrioid. This is an essential difference, as endometrioid cancers are the majority of endometrial cancers, most commonly present as early‐stage grade 1–2 disease, are often hormone dependent, and have a more favorable clinical course. Endometrioid cancers grade 3 are a more mixed entity and have in general a less favorable prognosis. Non‐endometrioid cancers comprise the more aggressive serous cancers, clear cell cancers, and carcinosarcomas, and these typically have higher risk of early distant spread and presentation in more advanced stage of disease.

Molecular profiling, according to The Cancer Genome Atlas (TCGA), points toward a paradigm shift from morphological to molecular classification.^3^ The TCGA studies have identified four molecular subgroups characterized, respectively, by *POLE* mutation (*POLE*mut group), microsatellite instability (mismatch repair deficient [MMRd] group), high somatic copy‐number alterations (serous‐like group, driven by *TP53* mutation, also called p53abn group), and a copy‐number low group without a specific driver mutation (NSMP group), each with a distinct prognosis.^3^, ^4^ The *POLE* mutated tumors, despite their aggressive appearance, have an extremely favorable prognosis, while the copy‐number high group driven by *TP53* mutation has an unfavorable prognosis. The prognosis of the mismatch repair deficient tumors and those with no specific molecular profile (NSMP) is relatively favorable, in between those with *POLE*mut and p53abn tumors. Several groups have shown that the TCGA molecular groups can be identified on formalin‐fixed paraffin‐embedded (FFPE) tissues using a surrogate marker approach: immunohistochemical markers for p53 and the mismatch repair proteins, and mutation analysis to detect pathogenic *POLE* mutations. This approach has been validated in several cohorts where the different TCGA groups indeed carry a similar prognosis.^5^, ^6^, ^7^, ^8^, ^9^ It should be noted that about 3% of the endometrial cancers have so‐called multiple classifying features, and their clinicopathological and molecular characteristics and outcome have recently been analyzed, supporting the classification of MMRd‐p53abn endometrial carcinomas as MMRd, and *POLE*mut‐p53abn endometrial carcinomas as *POLE*mut.^10^

Based on this more clinical approach, the TCGA classification groups have shown improved prognostic relevance and lack interobserver variability when compared to the historical morphological classification.

This molecular classification is likely the most innovative progress in the endometrial carcinoma field in recent years. Integration in daily practice is being implemented both in national and international guidelines^11^ and should be incorporated in diagnostics, treatment protocol, and future studies.

#### 1.3.2 Histopathologic grades (G)

- GX: Grade cannot be assessed
- G1: Well differentiated
- G2: Moderately differentiated
- G3: Poorly or undifferentiated.

Degree of differentiation of the adenocarcinoma is another basis for classifying carcinoma of the corpus, which is grouped as follows:

- G1: less than 5% of a nonsquamous or nonmorular solid growth pattern
- G2: 6%–50% of a nonsquamous or nonmorular solid growth pattern
- G3: greater than 50% of a nonsquamous or nonmorular solid growth pattern.

#### 1.3.3 Pathologic grading notes

A binary FIGO (International Federation of Gynecology and Obstetrics) grading is recommended, which considers grade 1 and grade 2 carcinomas as low grade and grade 3 carcinomas as high grade.^11^

Most authors consider serous and clear cell carcinomas high grade by definition.

Grading of adenocarcinomas with squamous differentiation is allocated according to the nuclear grade of the glandular component.

### 1.4 FIGO staging classification

Table 1 shows the current FIGO staging classification for cancer of the corpus uteri. Comparison of the stage groupings with the TNM classification is represented in Table 2.

**TABLE 1:** Cancer of the corpus uteri

FIGO stage	
I^a^	Tumor confined to the corpus uteri
IA^a^	No or less than half myometrial invasion
IB^a^	Invasion equal to or more than half of the myometrium
II^a^	Tumor invades cervical stroma, but does not extend beyond the uterus^b^
III^a^	Local and/or regional spread of the tumor
IIIA^a^	Tumor invades the serosa of the corpus uteri and/or adnexa^c^
IIIB^a^	Vaginal involvement and/or parametrial involvement^c^
IIIC^a^	Metastases to pelvic and/or para‐aortic lymph nodes^c^
IIIC1^a^	Positive pelvic nodes
IIIC2^a^	Positive para‐aortic nodes with or without positive pelvic lymph nodes
IV^a^	Tumor invades bladder and/or bowel mucosa, and/or distant metastases
IVA^a^	Tumor invasion of bladder and/or bowel mucosa
IVB^a^	Distant metastasis, including intra‐abdominal metastases and/or inguinal nodes

^a^ Either G1, G2, or G3.

^b^ Endocervical glandular involvement only should be considered as Stage I and no longer as Stage II.

^c^ Positive cytology has to be reported separately without changing the stage.

**TABLE 2:** Cancer of the corpus uteri: FIGO staging compared with the TNM classification^a^

FIGO Stage	Union for International Cancer Control (UICC)		
	T (tumor)	N (lymph nodes)	M (metastasis)
I	T1	N0	M0
IA	T1a	N0	M0
IB	T1b	N0	M0
II	T2	N0	M0
III	T3	N0–N1	M0
IIIA	T3a	N0	M0
IIIB	T3b	N0	M0
IIIC1	T1–T3	N1	M0
IIIC2	T1–T3	N1	M0
IVA	T4	Any N	M0
IVB	Any T	Any N	M1

^a^ Carcinosarcomas should be staged as carcinoma.

#### 1.4.1 Regional lymph nodes (N)

- NX: Regional lymph nodes cannot be assessed
- N0: No regional lymph node metastasis
- N1: Regional lymph node metastasis to pelvic lymph nodes
- N2: Regional lymph node metastasis to para‐aortic lymph nodes, with or without positive pelvic lymph nodes.

#### 1.4.2 Distant metastasis (M)

- MX: Distant metastasis cannot be assessed
- M0: No distant metastasis
- M1: Distant metastasis (includes metastasis to inguinal lymph nodes or intraperitoneal disease).

#### 1.4.3 Rules related to staging

During staging, distance from tumor to serosa should be measured. Other features should also be reported in the pathologic report of the hysterectomy specimen. Apart from the molecular classification, other traditional strong pathologic features, such as histopathologic type, grade, myometrial invasion, and lymphovascular space invasion (LVSI), are important in assessing prognosis. The presence of LVSI should also be indicated especially, as patients with LVSI‐positive tumors have a significantly worse prognosis, especially if extensive LVSI is found.^12^ Extensive or substantial LVSI is defined by multifocal or diffuse arrangement of LVSI or the presence of tumor cells in five or more lymphovascular spaces, in contrast to focal LVSI (presence of a single focus around the tumor).^13^, ^14^ The distinction made using LVSI status could be more relevant than the distinction between Stages IA and IB for predicting survival in Stage I endometrial cancer.^15^

As a minimum, any enlarged or suspicious lymph nodes should be removed in all patients. For high‐risk patients (grade 3, deep myometrial invasion, cervical extension, serous or clear cell histology), complete pelvic lymphadenectomy and resection of any enlarged para‐aortic nodes is recommended.

Clinical staging, as designated by FIGO in 1971, applies to a small percentage of corpus cancers that are primarily treated with radiation therapy or hormones due to patient factors. In those instances, the designation of that staging system should be noted.

## 2 INTRODUCTION

### 2.1 Incidence

Endometrial cancer represents the sixth most common malignant disorder worldwide. An estimated 382 000 new cases were diagnosed with this malignancy in 2018.^16^ High‐income countries have a greater incidence of endometrial cancer (11.1 per 100 000 females) compared with low‐resource countries (3.3 per 100 000 females). This might be attributable to high rates of obesity and physical inactivity—two major risk factors in high‐income countries, and to ageing of the population. Specifically, elevated estrogen levels are known to be the most likely cause of the increased risk of endometrial cancer for postmenopausal obese women.^17^ Conversely, physical activity and long‐term use of continuous combined estrogen–progestin therapy are associated with a reduced risk of endometrial cancer.^18^, ^19^ Interestingly, obesity is associated with earlier age at diagnosis, and with endometrioid‐type endometrial cancers. Similar associations were not observed with non‐endometrioid cancers, consistent with different pathways of tumorigenesis.^20^

North America and Europe have the highest incidence of endometrial cancer, where it is the most frequent cancer of the female genital tract and the fifth most common site in women after breast, lung, colorectal, and non‐basal skin cancer.^16^

According to the International Agency for Research on Cancer, the incidence rate of endometrial cancer is increasing rapidly and is estimated to increase by more than 50% worldwide by 2040.^21^ The incidence rates have been reported to have an increasing trend in the USA and several European countries since around 2000.^22^ The two major factors that contribute to an increase in the incidence of endometrial cancer in high‐income countries are increased prevalence of obesity and extended life expectancy. Other determinants—such as the widespread decrease in use of estrogen plus progestin menopausal hormone therapy—have also been proposed as the cause of the increased incidence rates of endometrial cancer in North America.^23^

Mortality rates of endometrial cancer showed a decrease in most European Union member states among women born before 1940. Improved cancer treatment and access to health care have been suggested as contributing to this decrease in cancer mortality.^24^

Conversely, the mortality caused by endometrial cancer had been found to be the highest among women of low socioeconomic status,^25^ possibly because of reduced evidence‐based care.^26^

### 2.2 Pathophysiology

Endometrial cancer research has gained some momentum in recent years and insights obtained from those studies have significant implications in the clinic. Endometrioid adenocarcinoma progresses through a premalignant phase of intraepithelial endometrial neoplasia in a large proportion of cases, such as hyperplasia with atypia.^27^ Most endometrial carcinomas arise as a result of a sequence of somatic DNA mutations, such as *PTEN*, mismatch repair genes, and *TP53* mutations. Mutations in the tumor suppressor *TP53* have been shown to play a pivotal role in serous endometrial cancer.^28^ Of note, human epidermal growth factor receptor 2 (HER2) amplification and homologous repair deficiency are also frequently found in this group.^29^, ^30^

Within the subgroup of mismatch repair deficient cancers, the most frequent cause of loss of expression of one or more of the mismatch repair genes is *MLH1* promotor hypermethylation, and other MMRd cancers are caused by double somatic hits. Lynch syndrome, a germline mutation of one of the mismatch repair genes, is found in 3% of all endometrial cancers, and in 10% of those with mismatch repair deficiency.^31^

### 2.3 Diagnosis

The utility of screening for endometrial cancer should be considered only in high‐risk populations.^32^ Transvaginal ultrasound is a possible screening test, as it is reasonably sensitive and specific. Screening can be considered for high‐risk groups, such as those with Lynch type 2 syndrome with a wish for fertility preservation, before the decision for prophylactic hysterectomy is made at a later age. In these cases, endometrial surveillance is performed by aspiration biopsy and transvaginal ultrasonography starting from the age of 35 years (annually until hysterectomy). Prophylactic surgery (hysterectomy and bilateral salpingo‐oophorectomy), preferably using a minimally invasive approach, should be discussed at the age of 40 years as an option for Lynch type 2 syndrome mutation carriers to prevent endometrial and ovarian cancer.^11^

After physical and pelvic examination, the first test to evaluate for signs of endometrial cancer is transvaginal ultrasound—an effective first test with a high negative predictive value when the endometrial thickness is less than 5 mm.^33^ Specifically, combination of transvaginal ultrasound with endometrial biopsies obtained by curettage has been shown to have a negative predictive value of 96%. When a biopsy is required, this can be obtained usually as an office procedure using a number of disposable instruments developed for this purpose. In patients with diagnostic uncertainty, hysteroscopy may be performed, and with flexible instruments can also be done without recourse to general anesthesia. However, the prognostic role of cells that are transtubally flushed during hysteroscopy remains uncertain. Anesthesia might be necessary in cases of cervical stenosis or if patient tolerance does not permit an office procedure. Individuals whose pelvic examination is unsatisfactory may also be evaluated with transvaginal or abdominal ultrasound to rule out concomitant adnexal pathology.

After a histopathologic diagnosis of endometrial adenocarcinoma, other factors need to be assessed. These include the local extent of the tumor, evidence of metastatic disease, as well as perioperative risk.

The pathology report from endometrial sampling should indicate at least the tumor type and grade of the lesion. Overall, there is only moderate agreement on tumor grade between preoperative endometrial sampling and final diagnosis, with the lowest agreement for grade 2 carcinomas, as grade is dependent on percentage of solid growth that can better be assessed in the final uterine specimen. Agreement between hysteroscopic biopsy and final diagnosis is higher than for dilatation and curettage; however, it is not significantly higher than for office endometrial biopsy.^34^

Full biochemistry (renal and liver function tests), and blood count also represent routine tests in the diagnosis of corpus uterine cancers. A chest X‐ray is often performed as it is a universally available, low‐cost examination and the consequences of detecting lung metastases, although rare in early‐stage disease, are significant. Serum CA125 may be of value in advanced disease for follow‐up. Evaluation for metastasis is useful particularly in patients with suspected advanced disease, non‐endometrioid histology, and for example in case of abnormal liver function tests. In high‐risk patients, CT‐based imaging of the chest, abdomen, and pelvis or PET‐CT may help determine the surgical approach. Cystoscopy and/or proctoscopy may be helpful if direct extension to the bladder or rectum is suspected.

## 3 PROGNOSTIC TUMOR CHARACTERISTICS FOR HIGH‐RISK DISEASE

Its early presentation following postmenopausal bleeding results in a generally good prognosis for endometrial cancer, but it should be treated using evidence‐based protocols and, where appropriate, by expert multidisciplinary teams. Four main histopathologic criteria are recommended to determine high‐risk disease:

- Tumor grade 3 (poorly differentiated)
- Lymphovascular space invasion (especially substantial/extensive LVSI)
- Non‐endometrioid histology (serous, clear cell, undifferentiated, small cell, carcinosarcoma)
- Cervical stromal involvement.

Since the molecular groups have been defined, the group of p53 abnormal cancers should be considered high risk; this risk is clearly higher than that of grade 3 or cervical stromal invasion. In a comprehensive analysis of grade 3 cancers, all four molecular subgroups were found and again the p53abn cancers had an unfavorable prognosis, while the *POLE* cancers had an excellent prognosis, and those with MMRd and NSMP in between.^35^ The importance of the molecular groups was subsequently confirmed in the molecular analysis of the PORTEC‐3 trial.^9^

It has been shown that, in the presence of the molecular groups, other main unfavorable factors such as substantial LVSI, L1 cell adhesion molecule (L1CAM) overexpression, and negative estrogen/progesterone receptors (ER/PR) can still contribute to prognostic information, and integrated risk profiles are promising for the clinic.^36^, ^37^

In a recent study of LVSI in a large Swedish population analysis, “obvious” LVSI was again confirmed as a very strong negative prognostic factor, and was associated with lymphatic spread and impaired survival even in the absence of lymph node metastases.^13^

L1CAM was introduced as a promising biomarker for identification of patients with poor outcome, which has been confirmed in subsequent studies.^37^, ^38^, ^39^ Markers of the p53 pathway,^28^ hormone receptor expression,^40^ and microsatellite instability^41^ are several of the other relevant biomarkers to predict prognosis of endometrial cancer. Various approaches combining genomic characterization and biomarkers expression provide promising results to tailor adjuvant therapy.^5^, ^8^, ^37^, ^42^

Also in the more molecular era, staging is recommended and based on traditional morphologic criteria.^11^ Based on the molecular studies, we know that the copy‐number high/p53 mutant/p53 abnormal genotype is more frequently diagnosed in high stage cancer.^3^, ^6^, ^8^, ^37^, ^43^ Prospective observational studies integrating surgical staging information with the genomic classification are recommended for refinement of surgical approach based on molecular and other risk factors.

MRI scanning and intraoperative frozen section represent the most accurate means of assessing both the depth of myometrial invasion and cervical involvement.^44^, ^45^, ^46^ Although CT and MRI are equivalent in terms of evaluating nodal metastases, neither is suitable to replace surgical lymph node assessment, which provides histological confirmation.^47^ PET‐CT is the best imaging method to evaluate lymph node and distant metastases, and could be considered in high‐risk or advanced stage disease.^48^ The role of PET‐MRI is currently being investigated but first evaluations support that it might provide an alternative diagnostic strategy to conventional imaging modalities in the preoperative staging of endometrial cancer.^49^

Nonsurgical staging of endometrial cancer, where extrauterine disease exists, is inherently inaccurate. This is particularly the case for the detection of small nodal involvement, intraperitoneal implants, and adnexal metastasis.

## 4 SURGICAL STAGING PROCEDURE FOR ENDOMETRIAL CANCER

Staging of endometrial cancer was changed from clinical to surgical in 1988 by the FIGO Gynecologic Oncology Committee. This recommendation has led to considerable debate and effort to define surgical staging procedures that can be implemented internationally. The traditional protocol included opening of the abdomen with a vertical midline incision and peritoneal washings taken immediately from the pelvis and abdomen, followed by careful exploration of the intra‐abdominal contents. The omentum, liver, peritoneal cul‐de‐sac, and adnexal surfaces should be examined and palpated for any possible metastases. These procedures should be followed by careful palpation for suspicious or enlarged nodes in the aortic and pelvic areas. However, laparoscopic procedures have increasingly been introduced as standard, especially for early‐stage disease, as these have been proven safe and reduce acute treatment‐related complications.^50^, ^51^, ^52^, ^53^ The recommended standard surgical procedure is an extra‐fascial total hysterectomy with bilateral salpingo‐oophorectomy. Adnexal removal is recommended even if the tubes and ovaries appear normal, as they may contain micrometastases. In premenopausal women with low‐grade, early‐stage disease, ovarian preservation could be considered.^54^, ^55^ Vaginal cuff removal is not advised, nor is there any benefit from excising parametrial tissue in the usual case. Where obvious cervical stromal involvement is demonstrated preoperatively, a modified radical hysterectomy has been historically performed. However, there is consensus that simple hysterectomy with free margins together with pelvic and para‐aortic lymphadenectomy may be sufficient.^11^, ^56^

Robot‐assisted surgery for the surgical treatment of patients suffering from early‐stage endometrial cancer is associated with favorable surgical and oncologic outcomes, particularly also for higher surgical risk groups such as elderly and obese women, thus permitting a low morbidity minimally invasive surgical approach for the majority of patients in expert centers.^57^, ^58^ In Denmark, the introduction of robotic surgery was associated with improved survival although causation remains to be proven.^59^

The utility of lymphadenectomy of the pelvic and para‐aortic areas is disputed, albeit it is currently mandated through the staging system.^60^ Currently, it is advised that complete lymphadenectomy is reserved for cases with high‐risk features. In contrast, selective node sampling has been deemed dubious as a routine approach. Since many individuals with endometrial cancer are obese or elderly, with concomitant medical problems, clinical judgment is required to determine if additional surgery is warranted. Any deeply invasive tumor or radiological suggestion of positive nodes is an indication for retroperitoneal lymph node evaluation, which might be followed by removal of any enlarged or suspicious nodes. Documentation of positive nodes identifies a high‐risk population and helps to tailor adjuvant treatment. Nodal resection also allows identification of node negative patients, potentially reducing the need for external beam radiotherapy.^11^

Several parameters advocate for aortic node resection. These include suspicious aortic or common iliac nodes, grossly positive adnexa, grossly positive pelvic nodes, and high‐grade tumors showing full thickness myometrial invasion. Patients with clear cell, papillary serous, or carcinosarcoma histologic subtypes are also candidates for aortic node resection.

A thorough preoperative assessment, with particular attention to the pathology and to radiological features has been defined as the most effective strategy for the triaging of these patients.^61^ Triaging for lymphadenectomy is also possible during surgery. Intraoperative assessment mainly involves assessment of myometrial invasion.^44^, ^45^ Grading on frozen section is possible, though suboptimal compared with preoperative grading.^45^

Concerning sentinel lymph node biopsy, several key surgical points should be respected^62^:

1. Expertise of the surgeon and attention to technical detail.
2. Superficial and deep cervical injection of dye.
3. Complete evaluation of the peritoneal cavity (sentinel lymph node mapping is for clinical Stage I, apparent uterine‐confined disease).
4. Sentinel lymph node dissection begins with evaluation of the retroperitoneal spaces and identification of the sentinel drainage pathways that emanate from the parametria, followed by excision of the most proximal lymph nodes in the sentinel pathway.
5. Any suspicious lymph nodes should be removed regardless of sentinel lymph node mapping and frozen section analysis may influence the decision to perform para‐aortic lymphadenectomy in some cases.
6. Performance of hemipelvic side‐specific lymphadenectomy for mapping failure has been shown to reduce false‐negative staging.
7. Enhanced pathology evaluation of sentinel lymph nodes with serial sectioning and immunohistochemistry stains increases the detection of low‐volume metastasis.

## 5 WHO SHOULD PERFORM THE SURGERY?

Full surgical staging is not required for low‐risk tumors, defined as well‐differentiated tumors with less than 50% myometrial invasion, with positive nodes in less than 5% of cases. Women with these tumors can be safely operated on by a general gynecologist. Patients at greater risk of extrauterine disease who may require lymphadenectomy should, in contrast, be operated on by gynecological oncologists. Care provided by gynecologic oncologists has been associated with better survival in high‐risk cancers^63^ and results in efficient use of healthcare resources and minimization of the potential morbidity associated with adjuvant radiation.^64^

## 6 WHEN SHOULD SURGERY BE PERFORMED?

The effect of waiting time for surgical staging on survival outcome for endometrial cancer is controversial. It has been suggested that a longer waiting time for surgical staging was associated with worse survival outcomes in uterine cancer^65^ and the delay between diagnosis and surgery should not exceed 6 weeks.^66^ However, when focusing on type 1 endometrial cancer only, the waiting time for surgical staging was not associated with decreased survival outcome, presumably owing to its indolent growth and resulting excellent prognosis.^67^

## 7 IS LYMPHADENECTOMY THERAPEUTIC?

Lymphadenectomy is required for accurate staging and is considered a staging procedure. Its potential therapeutic benefits are mainly contribution to accurate indication for adjuvant therapy. Historically, one case–control study suggested that lymphadenectomy may be beneficial therapeutically^68^ and another showed it improved prognosis even in node‐positive women.^69^ Another retrospective study suggested that complete lymphadenectomy increases survival in patients with grade 3 tumors.^70^ In contrast, two major trials of large‐scale cohorts have shown that pelvic lymphadenectomy offers no therapeutic benefits compared with no lymphadenectomy.^71^, ^72^ At present, lymphadenectomy is primarily used for staging and should be considered in women with high‐risk factors; however, sentinel lymph node biopsy is an acceptable alternative to systematic lymphadenectomy in early‐stage endometrial cancer.^11^ The ongoing ENGOT‐EN2‐DGCG trial (NCT01244789) aims to shed light into this issue by comparing survival with or without adjuvant chemotherapy in patients with Stage I grade 3 endometrioid endometrial cancer, Stage I and II type 2 endometrial cancer, or Stage II endometrioid endometrial cancer without metastatic nodes.

In a retrospective study, para‐aortic lymphadenectomy resulted in an improved outcome in intermediate and high‐risk patients when compared with pelvic lymphadenectomy alone.^73^ A limiting factor of this study was that adjuvant therapy was not comparable in the two groups. However, based on these findings, it is suggested that if lymphadenectomy is decided, both pelvic and para‐aortic infrarenal lymph node dissections are performed.

Sentinel lymph node mapping has been introduced into the surgical staging of endometrial cancer with the goal to reduce morbidity associated with comprehensive lymphadenectomy and to obtain prognostic information from lymph node status. The latest meta‐analysis reported an overall detection rate of 96%, with 73% bilateral pelvic node detection rate.^74^ Use of indocyanine green increases the bilateral detection rate compared with blue dye and is preferred.^75^ Additionally, cervical injection increases the bilateral sentinel lymph node detection rate but decreases the para‐aortic detection rate compared with alternative injection techniques. A meta‐analysis pooling approximately 6000 patients suggests that sentinel lymph node mapping is more targeted for less node dissection and more detection of positive lymph nodes even in high‐risk patients.^76^ Since sentinel lymph node mapping can safely replace lymphadenectomy in the staging of endometrial cancer, it is becoming the preferred method for lymph node sampling, even in high‐risk cancer. However, side‐specific systematic lymphadenectomy should be performed in high intermediate‐risk/high‐risk patients if the sentinel lymph node is not detected on either pelvic side. Pathological ultrastaging of sentinel lymph nodes is recommended.^11^

## 8 ADJUVANT TREATMENT

The indication for adjuvant radiation therapy is based on stage, tumor type, and the presence of risk factors including molecular factors.^36^ Low‐risk disease (Stage I, grade 1 or 2 with no or superficial myometrial invasion) does not require adjuvant radiation therapy. This was demonstrated in a Danish cohort study of low‐risk women, in which surgery alone resulted in a 96% 5‐year survival.^77^ In multiple randomized trials (PORTEC‐1 trial,^78^ the US GOG#99 trial,^79^ and the UK MRC ASTEC trial^80^), adjuvant pelvic radiation therapy was shown to significantly reduce the rates of vaginal and pelvic recurrence, but without overall survival benefit, while external beam radiation therapy (EBRT) added to the risk of long‐term morbidity. The patients without lymphadenectomy analyzed in the PORTEC and ASTEC trials presented similar recurrence and survival rates to those with documented node‐negative disease in the GOG#99 trial. Additionally, PORTEC‐1 illustrated that most pelvic relapses were located in the vaginal vault (75%), and that salvage rates were high in women who had not had previous radiation therapy.^81^

The PORTEC‐2 trial compared EBRT and vaginal brachytherapy in women with high/intermediate risk factors.^82^ This trial showed that vaginal brachytherapy had excellent vaginal control rates (<2% at 5 years for both EBRT and vaginal brachytherapy groups), with minimal adverse effects and significantly better quality of life. Quality of life of patients in the brachytherapy group remained the same as those of an age‐matched normal population.^83^ Since this seminal trial, vaginal brachytherapy has replaced EBRT as standard adjuvant treatment for patients with high/intermediate risk factors.

In a Danish study, omission of any EBRT or vaginal brachytherapy for high/intermediate risk disease led to higher recurrence rates (22% for intermediate risk disease, of which 15% was locoregional) without affecting survival rates,^84^ which has been confirmed by an analysis of survival in patients refusing adjuvant radiotherapy.^85^ A patient preference study showed that patients’ preferences are biased toward a treatment preventing relapse.^86^ Current knowledge on the molecular groups and other significant risk factors (LVSI, L1CAM) has led to the PORTEC‐4a trial, in which the role of an integrated molecular profile to determine adjuvant treatment, aimed at reducing both overtreatment and undertreatment, is compared with standard vaginal brachytherapy.^87^

Since adjuvant radiotherapy alone and adjuvant chemotherapy alone have shown similar impact on overall or relapse‐free survival in patients with endometrial cancer with risk factors or more advanced stages,^88^, ^89^ several studies have investigated the effect of the combination of chemotherapy and radiotherapy. A meta‐analysis pooling the results of two randomized trials (NSGO‐EC‐9501/EORTC‐55991 and MaNGO ILIADE‐III) investigating the therapeutic value of combining adjuvant platinum‐based chemotherapy with EBRT in patients with risk factors (grade 3 or deep invasion or adverse histologies) found a significant 9% improvement in progression‐free survival (69% vs 78% at 5 years; hazard ratio [HR] 0.63) with the addition of chemotherapy to EBRT, and a trend for a 7% improvement in 5‐year overall survival (75% vs 82%; HR 0.69, *P *= 0.07).^90^

More recently, the results of three large randomized trials (GOG#249, GOG#258, and PORTEC‐3) have been published. The randomized GOG‐249 trial, which recruited 601 patients with Stage I−II endometrial cancer with high/intermediate or high‐risk factors, compared vaginal brachytherapy plus three cycles of carboplatin‐paclitaxel chemotherapy with pelvic EBRT alone.^91^ The results showed no differences in relapse‐free survival between the arms, while there was better pelvic and peri‐aortic nodal control in the pelvic EBRT arm and more acute toxicity in the chemotherapy arm. It was concluded that for Stage I–II endometrial cancer with (high) risk features, pelvic EBRT is still the standard of care.^91^

In the PORTEC‐3 trial, patients with high‐risk Stage I−II or Stage II endometrial cancer (32% had grade 3, 29% serous or clear cell cancer, and 45% Stage III disease) were randomly allocated to pelvic EBRT alone or EBRT with two concurrent cycles of cisplatin in weeks one and four of EBRT, followed by four cycles of carboplatin and paclitaxel.^92^ In updated survival analysis at a median follow‐up of 72 months, there was a significant difference of 5% in overall survival between the arms (81% for chemoradiotherapy vs 76% for radiotherapy alone, *P *= 0.034), and a significant difference in failure‐free survival of 7% (76% vs 69%; *P *= 0.016).^92^ Women with Stage III disease had the highest absolute benefit of chemoradiotherapy, with 5‐year overall survival of 78% versus 68% for radiotherapy alone (*P *= 0.043). The large majority of recurrences were at distant sites (21% vs 29%) and pelvic recurrence was rare. In view of the toxicity of chemoradiotherapy with significantly more grade 3–4 adverse events during and after treatment and a persisting higher rate of grade 2 sensory neuropathy at longer term,^93^ it can be concluded that the combined schedule should primarily be recommended for women with serous cancers and those with Stage III disease.

In the randomized GOG‐258 trial for Stage III and Stage IV endometrial cancer (residual disease <2 cm allowed), 736 evaluable patients were randomized to receive either chemoradiotherapy (same schedule as used in PORTEC‐3 with two cycles of cisplatin during EBRT followed by four cycles of carboplatin and paclitaxel), or six cycles of carboplatin and paclitaxel alone.^94^ Addition of radiation therapy to chemotherapy did not improve overall (63% in both arms, not mature) or progression‐free survival (59% vs 58%), but the rate of pelvic and para‐aortic nodal relapse (11% vs 20%; HR 0.43) was significantly lower in the chemoradiotherapy arm. In the recently completed ENGOT‐EN2‐DGCG Phase 2 trial,^95^ patients with node‐negative endometrial cancer with high‐risk features were randomized to adjuvant chemotherapy (six cycles of carboplatin‐paclitaxel) or observation, with or without brachytherapy in both arms. This trial could add some answers to the questions regarding optimal use of adjuvant chemotherapy for women with high‐risk node‐negative endometrial cancer and results are awaited.

In the molecular analysis of the tumor tissues of 66% of the PORTEC‐3 trial participants, a statistically significant and clinically relevant survival advantage was found for p53abn carcinomas of all stages and, most notably, of all histologic subtypes treated with adjuvant chemoradiotherapy. In contrast, *POLE*mut carcinomas had almost no recurrences in both arms. There was benefit of added chemotherapy for MMRd, with overlapping overall and recurrence‐free survival curves for both arms, while the NSMP carcinomas had some benefit of chemoradiotherapy, especially in the case of Stage III.^9^ Prospective evaluation of the molecular characteristics and use of their specific properties in clinical trials is therefore highly recommended. Specifically, the MMRd cancers have been shown to have a strong CD8+ immune infiltrate and, in first studies, efficacy of checkpoint inhibition in metastatic MMRd cancers has been shown with response rates of about 43%.^96^

A subset of patients with p53abn disease harbors a HER2/NEU‐positive endometrial cancer (measured by overexpression or amplification). In this population, a recent randomized Phase 2 trial including 61 patients pointed toward an increased progression‐free survival and overall survival in women receiving trastuzumab in combination with paclitaxel‐carboplatin, with the greatest benefit seen for the treatment of Stage III–IV disease.^97^ Median progression‐free survival was 9.3 months versus 17.7 months among 41 patients with Stage III–IV disease undergoing primary treatment (HR 0.44). Another recent finding within the group of non‐endometrioid or p53abn cancers showed up to 50% having homologous recombination deficiency, suggesting a potential role of PARP inhibition.^29^ New studies incorporating these targeted drugs are being initiated.

In summary, adjuvant radiation therapy is not indicated in low‐risk patients and indicated in high‐risk patients. For patients with high/intermediate risk factors (at least two of the factors: age >60 years, deep myometrial invasion, grade 3, serous or clear cell histology, LVSI), vaginal brachytherapy alone is preferable to EBRT, providing excellent vaginal control without impacting quality of life. In patients with higher‐risk Stage I–II disease (grade 3 and deep invasion and/or LVSI, unfavorable histologies, unfavorable molecular factors), pelvic EBRT remains the standard of care. For p53abn and/or serous cancers of all stages, the use of adjuvant chemotherapy has been shown to provide survival benefit. Overall, the need for EBRT decreases when surgical staging identifies node‐negative disease.^11^ Surgical staging also allows clinicians to identify node‐positive (Stage III) disease that benefits from adjuvant therapy. For women with Stage III endometrial cancer, the combination of adjuvant chemotherapy and radiation therapy seems most effective to maximize recurrence‐free and overall survival. Ongoing and new studies with more individual assessment of molecular features will investigate their role in directing adjuvant treatment, and many new studies with emerging molecular targets are being initiated and the first are ongoing.

## 9 PROGESTOGEN THERAPY

Although the use of progesterone therapy has been widely recognized in the past, a meta‐analysis of six randomized trials totaling 3339 women has shown no survival benefit for adjuvant progestogen therapy in endometrial cancer.^98^ A subsequently published randomized trial of 1012 women also failed to demonstrate any survival benefit.^99^ However, hormonal therapy can provide prolonged remission of metastatic disease in women with grade 1 and/or ER/PR receptor‐positive disease. Where possible, ER/PR should be determined on a biopsy of the recurrent tumor because the hormone receptor status may change over time.^100^

An important other indication for progestogen therapy is to delay hysterectomy in case of early endometrial cancer diagnosed in young women wishing to preserve their fertility (see Section 13.3).

## 10 STAGE II

### 10.1 Occult Stage II disease

Therapeutic management of patients with clinically occult Stage II disease is similar to that of patients with Stage I disease.

### 10.2 Clinical overt Stage II disease

In case of macroscopic, bulky Stage II disease, radical hysterectomy, bilateral salpingo‐oophorectomy, bilateral pelvic lymphadenectomy, and selective aortic node dissection have been used as primary treatment. However, it is important to note that this strategy has been poorly supported by the medical literature. Results of one of the few retrospective studies could not find any survival benefit from radical hysterectomy for patients with suspected gross cervical involvement in comparison with simple or modified radical hysterectomy.^56^, ^101^ Surgical treatment in patients with suspected gross cervical involvement should be more radical only in order to operate with free surgical margins. Furthermore, this adapted type of hysterectomy is combined with full lymph node staging.^11^ Preoperative MRI scanning is advisable to exclude bladder involvement and ensure local resectability. Studies indicate excellent results for this approach, with no benefit from the addition of radiation for patients with negative nodes.^102^, ^103^ Adjuvant (chemo)radiotherapy is usually indicated depending on the risk factors (see Section 8 on adjuvant treatment).

In case of bulky disease, neoadjuvant therapy followed by a less extensive simple hysterectomy can represent an alternative. If surgery is not considered feasible because of tumor extension and/or in medically inoperable patients, full pelvic radiotherapy and intracavitary brachytherapy, as in cervical cancer, may be employed either preoperatively or definitively with high disease control and survival rates.^88^, ^89^

## 11 STAGE III

Most patients presenting with Stage III endometrial cancer are managed by complete surgical resection of all pelvic and/or nodal disease, followed by postoperative EBRT and/or chemotherapy.

As primary tumors of both the ovary and the endometrium may be present in patients with presumed Stage III disease with adnexal involvement, full surgical staging and expert pathologic examination of the specimen is recommended in these cases. Synchronous low‐grade endometrioid carcinomas of the endometrium and the ovary have been demonstrated mostly to be clonally related in the vast majority of cases. Their reported indolent behavior supports conservative management when the following criteria are met: (1) both tumors are low grade; (2) less than 50% myometrial invasion; (3) no involvement of any other site; (4) absence of extensive LVSI at any location.^11^

Adjuvant treatment is indicated for women with Stage III disease as detailed in Section 8.

Patients with clinical Stage III endometrial carcinoma in which surgical resection is not possible are treated primarily by pelvic irradiation, with or without chemotherapy, or alternatively by neoadjuvant chemotherapy, depending on the clinical situation.^11^, ^104^ Once therapy has been completed, exploratory laparotomy should be considered for those patients whose disease now appears to be resectable.

## 12 STAGE IV

Optimal management in women with Stage IV endometrial cancer with intraperitoneal disease only may include cytoreductive surgery, which is associated with superior overall survival outcome, especially when the metastatic sites are intra‐abdominal (peritoneum, omentum).^105^ In such advanced disease, neoadjuvant chemotherapy is also an option, particularly if postoperative morbidity is considered likely and/or ascites is present.^106^ A recent observational study including 102 patients showed that interval debulking surgery can be considered regardless of histologic subtype.^107^ In this population, progression‐free survival and overall survival depend on the amount of residual disease and the aim should be to leave no residual tumor. After surgery, platinum‐based chemotherapy should be considered, based on the trials cited above. Patients with evidence of extra‐abdominal metastases are usually managed with systemic platinum‐based chemotherapy, or hormonal therapy if grade 1 and/or receptor positive.

As neoadjuvant chemotherapy is the treatment of choice in advanced‐stage disease, as well as in relapsed disease, several studies have investigated the optimal combinations of chemotherapeutic agents that represent the most effective neoadjuvant therapy for Stage IV endometrial cancer patients. As the combination of doxorubicin, cisplatin, and paclitaxel (TAP)^108^ and carboplatin and paclitaxel have been shown to be most effective, these have been the most studied. The former, however, is much more toxic and resulted in treatment‐related deaths.

The carboplatin‐paclitaxel doublet has been tested in several Phase 2 studies in advanced‐stage or relapsed disease, demonstrating a response rate of 65%–75% and progression‐free survival of about 14 months.^109^, ^110^, ^111^ The results of the GOG‐0209 trial, a noninferiority trial in 1381 women comparing the combination of doxorubicin, cisplatin, and paclitaxel (TAP) with G‐CSF versus carboplatin and paclitaxel, showed that the carboplatin and paclitaxel doublet is noninferior to TAP.^112^ Better tolerability profile of carboplatin‐paclitaxel has led to the recommendation of the use of carboplatin and paclitaxel as the standard for adjuvant treatment in Stage III and IV disease.

Pelvic radiotherapy in Stage IV disease is sometimes considered to provide local tumor control. Similarly, symptomatic patients with vaginal bleeding or pain from a local tumor mass, or with leg edema due to lymph node involvement, are often palliated well with pelvic radiotherapy. Palliation of brain or bone metastases can be effectively obtained with short courses (1–5 fractions) of radiotherapy.

## 13 SPECIAL CONSIDERATIONS

### 13.1 Diagnosis post hysterectomy

Several therapeutic management problems have been reported to arise from diagnosis following hysterectomy. This is particularly true in cases where the adnexa have not been removed, which most often arises following vaginal hysterectomy for pelvic organ prolapse. Recommendations for further postoperative therapy are based on imaging (MRI and/or PET‐CT) and on known risk factors for extrauterine disease related to the histologic grade and depth of myometrial invasion. Individuals with grade 3 lesions, deep myometrial invasion, or LVSI may be candidates for additional surgery to remove the adnexa, and/or adjuvant EBRT. Patients with a grade 1 or 2 lesion with minimal myometrial invasion and no LVSI involvement generally require no further therapy.

### 13.2 Medically inoperable patients

The most common reasons for endometrial carcinoma to be deemed medically inoperable are morbid obesity and severe cardiopulmonary disease. In such cases, uterine brachytherapy is advised and has been shown to achieve cure rates in excess of 70%. In the presence of prognostic factors suggesting a high risk of involved nodes it can be combined with EBRT.^113^ Primary radiation therapy for medically inoperable patients with clinical Stage I and II endometrial adenocarcinoma provides disease control, with fewer than 16% of surviving patients experiencing recurrence.^114^

For patients with a well‐differentiated lesion, contraindications to general anesthesia, and who are unsuitable for radiotherapy, high‐dose progestins may be used. Trials using intrauterine hormone‐releasing devices instead of oral progestins are underway. In patients with contraindications to high‐dose progestins, the uterine hormone‐releasing device can be considered.

### 13.3 Diagnosis in young women

Since endometrial carcinoma is uncommon in women younger than 40 years, diagnosis during the reproductive years should be made with caution, and grade 1 endometrial carcinoma may be confused with severe atypical hyperplasia. In these women, consideration should be given to an estrogen‐related underlying condition such as a granulosa cell tumor, polycystic ovaries, or obesity. The safety of fertility preservation is well documented in grade 1 endometrioid endometrial cancer not invading the myometrium (as determined by MRI).^55^, ^115^ Progestins such as megestrol acetate (160–320 mg/day) or medroxyprogesterone acetate (400–600 mg/day) may be appropriate in these situations. Few studies reported the safety of fertility‐sparing management of grade 2 and 3 endometrial cancer.^116^ However, a large retrospective analysis reported an increased risk associated with uterine preservation in patients with grade 2 and 3 endometrial adenocarcinoma and suggested such management should be limited in time.^54^ Equivocal lesions should be examined by an experienced pathologist. In cases of complete response, conception must be encouraged and referral to a fertility clinic is recommended. Although the literature describes successful outcomes, fatal recurrences of endometrial cancer after a conservative approach have been reported; as such, the patient must be informed about the nonstandard treatment. Hysterectomy should be recommended once childbearing is complete.

Ovarian preservation, in patients with grade 1 intramucosal endometrial adenocarcinoma, might represent a beneficial therapeutic option, as this management was not associated with an increase in cancer‐related mortality in the largest sample available.^55^

## 14 FOLLOW‐UP

The objectives of follow‐up care for treated endometrial cancer patients are to provide information and psychological support, reassurance, evaluate and manage adverse effects from treatment, diagnose early recurrence, and collect data. The clinical and cost‐effectiveness of follow‐up implementation has been addressed internationally in one prospective^117^ and several retrospective studies.^118^, ^119^, ^120^ Overall, these studies found that about 75% of recurrences in endometrial cancer patients are symptomatic and 25% asymptomatic. Neither recurrence‐free nor overall survival was improved in asymptomatic cases compared with those detected at clinical presentation. Most (65%–85%) recurrences were diagnosed within 3 years of primary treatment, and 40% of recurrences were local. Another important finding of those studies was that the use of routine follow‐up Pap smears and chest X‐rays is not cost‐effective. Given the high salvage rate following radiotherapy, it has been suggested that nonirradiated patients are a group that would benefit from regular follow‐up to detect early vaginal recurrence.^121^

Two systematic reviews^122^, ^123^ documented evidence for the utility of follow‐up examinations, and concluded that follow‐up should be practical and directed by symptoms and pelvic examination. These studies also recommend reduction in the frequency of follow‐up visits for low‐risk patients. Given the low risk of recurrence, vaginal cytology can be omitted, resulting in reduced healthcare costs.^124^ It appears that visual inspection is sufficient, since positive cytology is merely diagnosed in cases of symptomatic recurrence.^120^, ^125^, ^126^

More recently, studies of minimal follow‐up (nurse led, telephone based) after the first year have been done and results are awaited.^127^, ^128^, ^129^ First results suggest good patient acceptability once prompt access to evaluation in case of symptoms is ensured.

Follow‐up care should also include patient counseling as these patients are at risk of second cancers following their primary endometrial cancer. For instance, the estimated incidence rate of Lynch syndrome in an unselected endometrial cancer population is 3%−6%.^130^ Routine pathologic screening of mismatch repair deficiency in the endometrial cancer specimen, similar to colorectal cancer, has been advocated and is increasingly being introduced in practice.^131^ However, in most women with mismatch repair deficiency this is caused by MLH1 promoter hypermethylation and a test of this before referring a patient to a clinical geneticist is recommended.^31^ Survivors of endometrial cancer have a three‐fold increased risk of second cancer when compared with a matched population. This risk increase seems mainly related to lifestyle factors and genetic susceptibility.^132^ These women should be counseled on exercise and weight loss programs.

## 15 RECURRENCE

The therapeutic management for localized recurrences includes surgery, radiation therapy, or a combination of both. The choice of these strategies depends on the primary therapy. Screening for distant metastases should be performed before deciding on curative treatment. If primary therapy consisted of surgery alone, radiotherapy represents an effective salvage strategy in cases of vaginal or central pelvic recurrence. In these cases, a combination of EBRT and brachytherapy, preferably image guided, is usually required. Large recurrences should be evaluated for excision, followed by radiotherapy. Alternatively, chemotherapy may be considered to decrease the volume of the recurrence and hence improve the chances of complete surgical resection. Additional chemotherapy with radiotherapy is being evaluated in a recently completed GOG trial (GOG‐0238, NCT00492778).^133^ Extended surgery may be justified, especially in patients who have had prior radiation therapy. However, radical surgery within irradiated fields (especially in the case of sidewall recurrence) frequently results in significant morbidity, such as treatment‐resistant pain and fistula formation. The results of pelvic exenteration in properly selected cases (central recurrences without signs of distant spread) are similar to those obtained in cervical cancer. Overall, survival rates in well‐selected patients are in the order of 50%.

Nonlocalized recurrent tumors of low grade and/or with positive hormone receptors are usually treated with progestin therapy: medroxyprogesterone acetate 50–100 mg three times a day or megestrol acetate 80 mg 2–3 times a day. Treatment is continued as long as the disease is stable or in remission. Maximum clinical response may only be observed three or more months after therapy initiation. Platinum‐based chemotherapy (cisplatin and doxorubicin, or carboplatin and paclitaxel) has been recommended for patients with advanced or recurrent disease, not amenable to cure by surgery and/or radiotherapy.^109^, ^134^ Several ongoing trials are currently investigating the clinical applicability of targeted therapies in patients with nonlocalized recurrent tumors, especially checkpoint inhibition, both given with and after chemotherapy and (in more recent studies) also as single agents or combinations compared with chemotherapy. Most of these ongoing studies and studies in set‐up are listed on the websites of the Gynecologic Cancer InterGroup (https://gcigtrials.org) and ENGOT (European Network of Gynaecological Oncological Trial groups (https://engot.esgo.org/).

## 16 RECOMMENDATIONS FOR PRACTICE

1. A definitive tissue diagnosis must be obtained preoperatively. This will result in better selection of the surgical approach and help to differentiate tumors at low‐ and high‐risk of lymph node metastasis. Imaging might be used to determine depth of myometrial invasion, cervical involvement, and lymph node enlargement. **Level of Evidence C**
2. Although lymphadenectomy in clinical Stage I endometrial cancer is required for staging purposes, it has no impact on overall or relapse‐free survival. **Level of Evidence A**. In the clinic, lymphadenectomy should be performed for staging only in high‐risk cases. There is little evidence to support a therapeutic benefit, but it may be used to select women with positive nodes for adjuvant therapy and reduce the need for EBRT in node‐negative patients. **Level of Evidence C**
3. In patients with Stage I endometrial cancer with intermediate or high/intermediate risk features, adjuvant radiotherapy has no impact on survival, but significantly reduces the rate of pelvic and para‐aortic recurrence. **Level of Evidence A**. In high‐risk patients, vaginal brachytherapy effectively reduces the risk of vaginal relapse. **Level of Evidence A**. EBRT should be considered in patients with presumed Stage I–II disease with strong adverse factors, positive nodes, or advanced stage disease to ensure pelvic control. **Level of Evidence A**
4. The addition of adjuvant chemotherapy to radiotherapy in patients with high‐risk disease improves progression‐free and overall survival. **Level of Evidence A**
5. Adjuvant chemoradiotherapy for patients with early stage, high‐risk disease should be considered for those with serous and/or p53abn cancers. **Level of Evidence B**
6. Adjuvant radiotherapy alone provides similar recurrence‐free survival compared to three cycles of adjuvant chemotherapy and vaginal brachytherapy, with lower risk of pelvic and peri‐aortic nodal recurrence. **Level of Evidence A**
7. Adjuvant chemoradiotherapy or adjuvant chemotherapy alone should be considered in patients with Stage III‐IV disease and abdominal disease with residual nodules less than 2 cm diameter. **Level of Evidence A**
8. Targeted therapy in endometrial cancer is being developed and participation in clinical trials is encouraged. **Professional consensus**
9. The use of adjuvant hormonal therapy (progestogen) has not been properly substantiated. **Level of Evidence A**
10. High‐risk and advanced‐stage endometrial cancer patients should be managed where possible by a gynecologic oncologist, working within a multidisciplinary team. **Level of Evidence A**
11. Patients with endometrial cancer are frequently old and frail, and this should be taken into consideration when prescribing adjuvant therapy. **Professional consensus**

## AUTHOR CONTRIBUTIONS

All authors contributed equally to this manuscript.

## CONFLICTS OF INTEREST

Outside of the submitted work, MK reports receipt of travel fees for surgical training from Intuitive Surgical. The other authors report no conflicts of interest.
